# Synergistic Signal Amplification via Weak Value Amplification Effect and Sandwich Structure for Highly Sensitive and Specific Real-Time Detection of CA125

**DOI:** 10.3390/bios15050268

**Published:** 2025-04-23

**Authors:** Bei Wang, Yang Xu, Han Li, Zishuo Song, Tian Guan, Yonghong He

**Affiliations:** 1 Shenzhen Key Laboratory for Minimal Invasive Medical Technologies, Institute of Optical Imaging and Sensing, Tsinghua Shenzhen International Graduate School, Tsinghua University, Shenzhen 518055, China; wangbei22@mails.tsinghua.edu.cn (B.W.); han-li22@mails.tsinghua.edu.cn (H.L.); songzs1998@stu.gxnu.edu.cn (Z.S.); 2 Institute of Biopharmaceutical and Health Engineering, Tsinghua Shenzhen International Graduate School, Tsinghua University, Shenzhen 518055, China; 3 Department of Laboratory Medicine, Shenzhen Children’s Hospital, Shenzhen 518038, China; wdxylioo@gmail.com

**Keywords:** aptamer, antibody, sandwich structure, weak value amplification, signal amplification

## Abstract

Biomolecule detection is pivotal in disease diagnosis. In this study, we present a novel aptamer–antibody sandwich module integrated with an imaging weak measurement system to enhance the sensitivity and specificity of biomolecule detection. The feasibility of this approach is demonstrated using CA125. CA125 is a glycoprotein tumor marker widely used for ovarian cancer diagnosis and monitoring, with its level changes closely associated with disease progression. Given its clinical significance, developing highly sensitive and specific CA125 detection methods is crucial for precision medicine. The dual-recognition mechanism combines the high affinity of aptamers and the specificity of antibodies, significantly improving detection performance while utilizing antibodies for signal amplification. In the presence of CA125, the anti-CA125 aptamer immobilized on the chip surface captures the target, which is then specifically bound by the CA125 antibody, forming the aptamer–CA125–antibody complex. This interaction induces a change in the refractive index of the chip surface, which is detected by the imaging weak measurement system and ultimately manifested as a variation in light intensity in the resulting images. The method achieves the highly sensitive detection of CA125 in the 0.01 mU/mL range to 100 U/mL, with preliminary results showing a detection resolution of 3.98 μU/mL and high specificity against non-target proteins. Additionally, detecting CA125 in serum samples further validates the feasibility of the method’s applicability in complex biological matrices. The proposed method offers significant advantages, including high sensitivity, high specificity, label-free, multiplexed detection, low cost, and real-time detection, making it a promising platform for bio-molecule detection with a wide range of applications.

## 1. Introduction

Biomolecular assays are fundamental tools in various fields, including life sciences, medical diagnostics, and environmental monitoring, providing critical insights into biological systems and offering solutions to complex real-world challenges. Biomolecules, such as proteins, nucleic acids, and small-molecule metabolites, play central roles in biological processes, and variations in their concentrations or functional states can serve as indicators of physiopathological conditions related to health and disease [1]. For example, carbohydrate antigen 125 (CA125) is a crucial tumor marker widely used for ovarian cancer monitoring and diagnosis, with its level fluctuations serving as indicators of disease progression, treatment response, and postoperative recurrence risk. Therefore, developing biomolecular detection technologies with high sensitivity and specificity is essential for advancing disease mechanism research and biomarker discovery and enabling the progress of precision medicine [2,3]. Recently, as complex biological samples have become more widely used, the demand for detection technologies that offer high sensitivity, specificity, and ubiquity has grown significantly.

Current biomolecule detection methods primarily rely on molecular recognition techniques, with antibodies and aptamers being two commonly used recognition elements [4,5,6]. Antibody-based methods, such as enzyme-linked immunosorbent assays (ELISA), exploit the specific binding between antibodies and target molecules to facilitate the efficient detection of proteins and other biomolecules [7,8]. In the clinical detection of CA125, ELISA and chemiluminescence are widely used methods, which play an important role in the diagnosis and monitoring of ovarian cancer. However, challenges such as the complexity of antibody preparation, difficulties in ensuring consistent quality, and the potential for spatial disruption of binding sites in multiple assays can limit their effectiveness in certain practical applications [9,10]. In contrast, aptamers—artificially selected single-stranded nucleic acid molecules—offer several advantages, including high stability, ease of chemical modification, and a broad range of available sequences [11]. Aptamers exhibit excellent recognition capabilities in protein, nucleic acid, and small molecule assays [12,13,14,15]. Aptamers targeting CA125 have gained increasing attention in recent years, providing a novel detection strategy. Many researchers have proposed various aptamer-based biosensing approaches for CA125 detection [16,17]. Nevertheless, their binding efficiency may decrease in complex biological sample environments, and background noise can increase, limiting their detection performance. Therefore, exploring innovative and efficient detection strategies has become a key focus in enhancing the detection performance of CA125 and other similar targets. Researchers have proposed the aptamer–antibody sandwich structure, leveraging the advantages of both aptamers and antibodies to ensure efficient target molecule capture and signal amplification [18]. Compared to traditional single-recognition-element methods, the sandwich structure significantly reduces background noise and enhances the detection signals, enabling highly specific and sensitive biomolecular detection. The application of the aptamer–antibody sandwich method has significantly improved the sensitivity of biomarker detection, and prior research in this direction provides strong support for the effectiveness of our approach [19,20,21].

There has been growing interest in developing new detection techniques to meet the increasing demand for higher sensitivity in recent years. Weak measurement, a concept rooted in quantum mechanical measurement theory, was first introduced by Aharonov et al. [22], while the existence of weak values was experimentally demonstrated by Ritchie et al. [23]. Unlike traditional quantum measurement methods, weak measurement relies on weak coupling and pre- and post-selection strategies, enabling the amplification of weak signals without altering the intrinsic properties of the target signal while effectively suppressing background noise [24,25]. Specifically, weak measurement can amplify weak signals through carefully designed weak coupling and post-selection processes, and the system’s measurement results can be anomalously enhanced by weak value [23,26]. This phenomenon is called weak value amplification (WVA). In 2015, He’s group realized the first frequency-domain weak measurement system with broad applicability, enabling the real-time monitoring of biomolecular binding processes [27]. It marked the first application of a weak measurement system in biomedical engineering. As weak measurement technology has evolved, it has been widely adopted across various fields, including quantum sensing [28,29,30], optical signal processing [31,32], biomedical detection [33,34,35,36], and quantum information processing [37,38], due to its unique advantages. In recent years, analytical detection methods incorporating weak measurement technology have been applied to protein–antibody interactions [34,36,39], small molecule detection [40], cellular protein analysis [41], and high-sensitivity detection of viral proteins [42], demonstrating superior signal amplification capabilities in complex sample environments. Compared to other detection methods, weak measurement not only enhances detection sensitivity but also effectively reduces background noise interference, thereby improving detection reliability. As weak measurement technology continues to advance, its applications in biomedical detection are expanding, offering an efficient approach for early disease diagnosis, personalized treatment, and biomolecular interaction studies.

**Figure 1:**
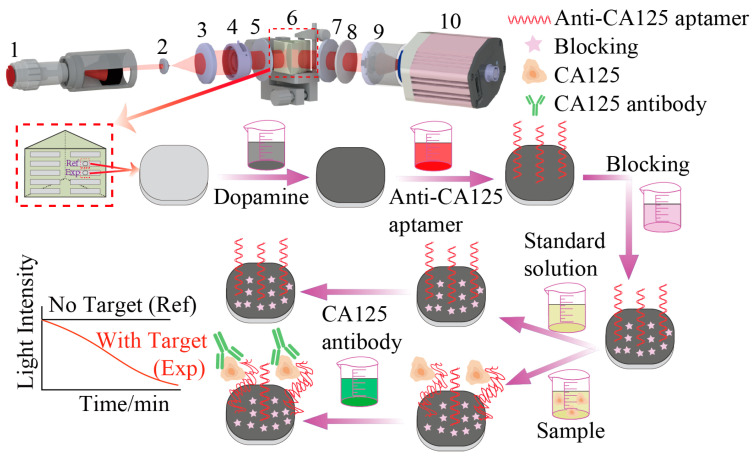
Schematic diagram of the principle of aptamer–antibody sandwich assay based on imaging weak measurement system. The system comprises the following components, labeled 1 to 10: 1—collimated light source; 2 and 3—a pair of beam expanding lenses; 4—diaphragm; 5—front polarizer; 6—sensing chip; 7—quarter-wave plate; 8—rear polarizer; 9—imaging lens; and 10—camera. The detection process primarily involves the functionalization of the sensing chip and the molecular detection procedures.

In this study, we propose a novel sensing strategy that combines the weak value amplification effect with aptamer–antibody sandwich structure, utilizing an imaging weak measurement system to achieve highly sensitive and specific biomolecular detection. Glycoprotein CA125, a biomarker for ovarian cancer, was selected as a model for validation, and the experimental design is illustrated in Figure 1. The homemade imaging weak measurement system utilized a camera for data acquisition and a 3D-printed chip channel as the reaction unit (Figure S1). The chip surface was pre-modified with dopamine to immobilize anti-CA125 aptamers. Upon introducing the target protein CA125, it was captured by the surface bound aptamers, forming an anti-CA125 aptamer–CA125 complex. Subsequently, the CA125 antibody was introduced, binding to the CA125 and completing the aptamer–CA125–antibody sandwich structure on the chip surface. Real-time experimental images captured by the camera recorded changes in light intensity, corresponding to variations in the refractive index of the chip surface caused by the molecular interactions. These light intensity changes, proportional to the concentration of CA125, were used as the detection signals. Antibodies provide high affinity and specific recognition, while aptamers offer excellent chemical stability, modifiability, and lower synthesis costs. Their dual recognition enhances both the specificity and sensitivity of detection, reduces nonspecific binding, and improves signal amplification and real-time detection capabilities, thereby increasing the reliability and application potential of the detection methods. Unlike traditional methods, the approach is label-free, enables real-time detection, and offers high accuracy. It effectively addresses the limitations of sensitivity and adaptability in complex sample environments, providing a promising platform for advanced biomolecular diagnostics and related applications.

## 2. Materials and Methods

### 2.1. Imaging Weak Measurement System and Detection Principles

Weak measurement is an extension of quantum measurement theory, first proposed by Aharonov et al. in 1988 [22]. Unlike traditional strong measurement, weak measurement employs a weakly coupled measuring device, causing only minimal disturbance to the system and preventing wave function collapse while still allowing the extraction of quantum interference information. At its core is the concept of the weak value, where measurement results can exceed the eigenvalue range under specific post-selection conditions. The weak value amplification effect leverages this property to enhance measurement sensitivity by precisely tuning the pre- and post-selected states, thereby amplifying weak signals. The approach has been widely applied in high-precision measurement fields such as optical interferometry, nanoscale displacement detection, and biosensing.

Optical methods are widely employed in the construction of weak measurement systems. The imaging weak measurement system used in this thesis utilizes the orthogonal polarization state of the light beam as the eigenstate and the dynamic longitudinal phase of photons as the pointer state, enabling interaction analysis through variations in photon count. The previously proposed weak measurement system based on the spectrometer is limited by the characteristics of the spectrometer and mainly relies on single-point analysis for detection, making it difficult to meet the demands of rapid and high-throughput detection. In this study, we use each detection unit of photodetector instead of the spectrometer as measurement device. Each detection unit of the photodetector enables photon counting (correlated with light intensity), allowing the simultaneous detection of multiple regions of interest on the reaction interface. Moreover, the abundance of detection units facilitates the construction of self-referential signals, effectively reducing noise and enhancing resistance to interference during bio-detection. This approach significantly extends the applicability of frequency-domain weak measurement technology to high-throughput biomolecules detection.

The configuration of the imaging weak measurement system is illustrated in Figure 1. The process begins with a collimated light source, whose beam sequentially passes through two beam expanding lenses to optimize beam quality and ensure uniform light intensity, which is an essential step for achieving high sensitivity and high-throughput detection in subsequent experiments. Next, a diaphragm can be introduced into the system to adjust the detectable range of the sensing chip. Its inclusion is optional and can be determined based on specific experimental requirements. The beam then enters the front polarizer, which performs the pre-selection process. Upon exiting the front polarizer, the beam passes through a ZF6 prism (refractive index = 1.73), where total internal reflection occurs at a specific incident angle. Using the prism as coupling substrate, the incident light satisfies the total internal reflection condition on the inner surface of the prism, generating an evanescent wave on the detection surface. This wave enables the ultrasensitive detection of molecular interactions occurring on the prism’s surface. The molecular interactions alter the effective refractive index of the prism surface, and this change in refractive index subsequently affects the phase difference between the p- and s-polarized light. After placing an achromatic quarter-wave plate behind the prism, the modulation process can convert the phase difference into a manipulable spin parameter. This critical modulation step transforms the otherwise challenging-to-measure phase difference into a measurable quantity. The modulated beam then passes through the rear polarizer, which makes the relationship between the polarization information and the spin parameter more explicit and detectable. Finally, the imaging lens focuses on the processed beam, capturing the total internal reflection interface with a camera for data acquisition. The theoretical principles underpinning this system are elaborated in detail in the Supporting Information (SI). The system’s performance was validated through experiments conducted with sodium chloride solutions at various concentrations, with the results presented in Figure S2.

The imaging weak measurement system used in this study can be regarded as an adaptation of an ellipsometry-based structure. While ellipsometry is solely focused on polarization detection, weak measurement, due to the presence of weak coupling, facilitates signal amplification. Imaging weak measurement system can amplify light intensity signals, thereby enhancing the detection of biological signals. These biological signals arise from the interaction between biomolecules (e.g., proteins) and their specific receptors, such as antibodies or aptamers. In our method, changes in the biosignal cause changes in the refractive index within the response region on the chip surface, and these refractive index changes are manifested as changes in the light intensity of the acquired image. This correspondence effectively combines the detection of biosignals with refractive index measurement and light intensity change detection and realizes the conversion of biosignal detection to light intensity detection. To illustrate, we take glycoprotein CA125 as an example. When an anti-CA125 aptamer immobilized on the chip surface captures the CA125 protein through specific binding, the interaction changes the reactive region’s refractive index. This change is observed as a variation in the light intensity in the captured image. Similarly, when CA125 interacts specifically with its antibody to form a complex, the refractive index shifts again, leading to another detectable change in light intensity. The imaging weak measurement system amplifies these refractive index changes, thereby enhancing the biological signal detection and enabling highly sensitive detection of CA125. The rest of the experiment-related content presentation is shown in the Supporting Information.

In this study, the prism surface was first modified with dopamine, followed by the immobilization of aptamers via the polydopamine membrane. The aptamer specifically recognizes the target protein, which subsequently binds to an antibody, forming an aptamer–protein–antibody complex. Since these molecular interactions primarily occur at the surface and do not induce significant cross-linking or stacking effects, we infer that the observed refractive index changes are mainly associated with the formation of a monolayer molecular structure. During the experiment, the formation of this monolayer alters the effective refractive index at the prism’s surface, thereby modulating the phase difference between p- and s-polarized light. By employing the weak measurement system, we monitored these refractive index variations in real time, demonstrating the potential of the approach for high-sensitivity biomolecular detection.

### 2.2. Experimental Procedures

For details of the procedures, see the relevant description in the Supporting Information. In the specific experiments of this study, only the concentration of the aptamer and different aptamer sequences were optimized, while all other experimental conditions followed standard baseline conditions. The selection of baseline experimental conditions was determined based on references and experimental experiences, and specific information and references are listed in the Supporting Information.

## 3. Results and Discussion

### 3.1. Weak Measurement Characterization

**Figure 2:**
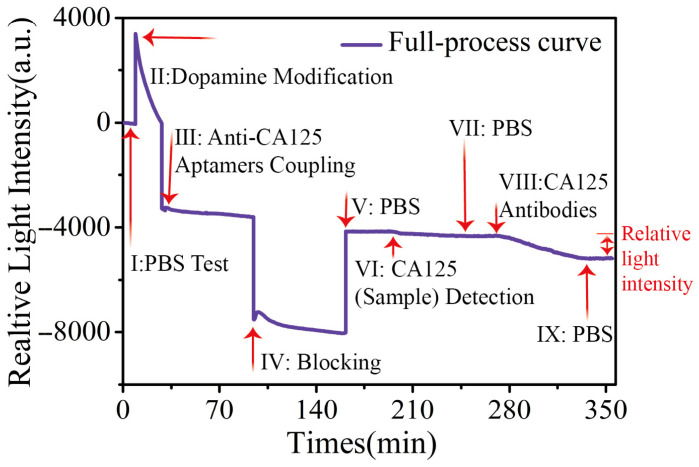
Changes in relative light intensity throughout the experimental process. (I) PBS test; (II) Dopamine: surface modification of the chip surface; (III) Aminated anti-CA125 aptamer immobilization; (IV) 2% blocking solution: blocking of blank sites; (V) PBS: calibration of the imaging weak measurement system; (VI) Glycoprotein CA125: sample detection; (VII) PBS: washing of unreacted CA125; (VIII) CA125 antibody: specific binding and formation of the sandwich structure; (IX) PBS: washing and end of the reaction.

The experimental procedure involves both the surface functionalization of the chip and the subsequent specific reaction processes, with CA125 detection serving as a representative example. Surface functionalization includes dopamine modification, aptamer immobilization, and the blockage of blank sites. Initially, a uniform dopamine film was deposited on the chip surface, followed by the coupling of the aminated anti-CA125 aptamer to the surface. Finally, the remaining blank sites on the chip surface were blocked to minimize non-specific interactions. The surface functionalization process is illustrated in steps I–IV in Figure 2. Step I involves passing the PBS solution through the channel for 10 min. This step cleans the flow channel and assesses the system’s stability. Since PBS does not bind to the chip surface, the real-time response curve of the weak measurement system remains smooth, with minimal fluctuations during this phase. Step II involves the dopamine modification process, where a dopaminen-tris solution is introduced into the channel and allowed to react for 20 min. Due to the refractive index difference between the dopamine solution and PBS, a sharp peak appears in the reaction curve. As the reaction progresses, dopamine undergoes self-polymerization in the alkaline environment, forming a polydopamine film on the chip surface. This results in a change in the refractive index of the surface, which is reflected as a variation in light intensity. The polydopamine film exhibits excellent adhesion properties and contains a variety of functional groups, such as hydroxyl, amino, and phenolic hydroxyl groups, which allow for the further chemical modification or binding with other materials. The adhesive properties and abundant functional groups of polydopamine allow aptamers to be immobilized on the prism surface through various mechanisms, including covalent binding, non-covalent interactions, or self-assembly. The properties of polydopamine enable step III, in which the aminated anti-CA125 aptamer is immobilized onto the chip surface via the polydopamine film. Although a significant amount of anti-CA125 aptamer is immobilized on the prism surface, due to factors such as surface space limitations, surface chemistry, the properties of the polydopamine film, aptamer concentration, and binding kinetics, the aptamers cannot occupy all available sites on the prism surface. Therefore, it is necessary to block the unreacted sites. Step IV involves sealing the blank sites with a 2% blocking solution to minimize the potential for non-specific signals. After completing the surface functionalization, the chips can be stored for future use.

The specific reaction process is illustrated in steps V–IX in Figure 2. Before initiating the specific reaction, a baseline calibration was conducted using PBS as a standard solution (Step V). Once the system reached a steady state, two specific binding events involving CA125 were carried out (Steps VI–VIII). The specific reaction process involves two steps: first, the binding of the anti-CA125 aptamer with CA125 (Step VI), followed by the specific interaction between CA125 and its antibody (Step VIII). These two binding events occur sequentially, forming a sandwich complex comprising the anti-CA125 aptamer, CA125 protein, and CA125 antibody. As shown in the curve in Figure 2, the PBS solution is introduced both before and after the molecular interaction process (aptamer recognizing CA125, CA125 protein reacting with its antibody). On the one hand, it serves to test the stability of the system, and on the other hand, it serves to wash away any excess molecules that did not participate in the reaction. Since the fluctuations during the introduction of the PBS solution are relatively stable, the difference between the two PBS segments can be considered as the relative change in light intensity caused by the molecular interaction. The detection of this sandwich complex serves as the experimental signal for data analysis. Figure 2 illustrates the schematic diagram of the reaction and the corresponding relative light intensity changes. The experimental results indicate that the method proposed in this study enables the highly sensitive detection of CA125.

### 3.2. Optimization of Anti-CA125 Aptamer Concentration

**Figure 3:**
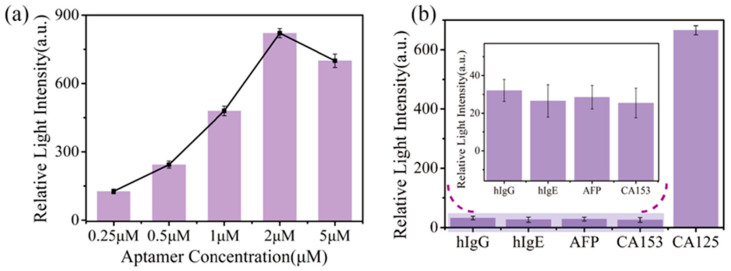
(**a**) Results of aptamer concentration optimization: Various concentrations of anti-CA125 aptamer were immobilized on the chip surface. Error bars represent the relative standard deviation from three independent experiments. (**b**) Specificity validation: human IgG, human IgE, AFP, and CA153 were used as interfering proteins to assess the specificity of the sensor for the target analyte.

The concentration of the anti-CA125 aptamer solution was optimized in this study. Throughout the experiment, all conditions were kept constant except for the variation in the concentration of the anti-CA125 aptamer. In the optimization experiments, we fixed the concentration of CA125 at 100 U/mL and varied the concentrations of the anti-CA125 aptamer to 0.25 μM, 0.5 μM, 1 μM, 2 μM, and 5 μM. After completing the coupling experiments, we washed the flow channels with PBS buffer to remove unreacted aptamers. We then carried out the sample assay following the specific reaction process, and statistically analyzed the light intensity changes. As shown in Figure 3a, the highest response signal was obtained at a concentration of 2 μM of anti-CA125 aptamer, demonstrating superior binding efficiency compared to other concentrations. It suggests that, at this concentration, the aptamer effectively occupies the dopamine-modified active sites on the chip surface, facilitating optimal specific binding. When the concentration of anti-CA125 aptamer was below 2 μM, the active sites were not fully utilized due to insufficient aptamer molecules, leading to a reduction in signal intensity. Conversely, when the concentration exceeded 2 μM, the active sites approached saturation; however, the higher concentration potentially caused non-specific adsorption, increased background noise, and yielded no significant enhancement in signal intensity. Additionally, excessive concentrations may lead to reagent wastage or alterations in solution properties, potentially impacting the experimental outcomes. Therefore, based on the optimization analysis, 2 μM was determined to be the optimal concentration of anti-CA125 aptamer for subsequent experiments. This concentration ensures the efficient binding and signal output while avoiding the limitations associated with lower concentrations and the potential side effects of higher concentrations, thereby facilitating the reliable progression of the experiments.

### 3.3. Specific Detection of CA125 Based on the Sandwich Method

Complex biological samples often contain diverse components. Ensuring specificity is a critical step in enabling the detection of complex samples using the imaging weak measurement-based sandwich method proposed in this study. To address this, specificity validation experiments were designed to evaluate the method’s ability to detect the target analyte selectively. Four interfering proteins—human IgG (hIgG), human IgE (hIgE), alpha-fetoprotein (AFP), and CA153—were employed in the specificity validation experiments. The concentrations of human IgG, IgE, and AFP were each set at 25 ng/mL, while the concentration of CA153 was 25 U/mL. These interfering proteins were introduced into the chip channel instead of CA125 to bind with anti-CA125 aptamer and then interacted with the CA125 antibody. The assay results are presented in Figure 3b. Each bar represents the mean response signal (corresponding to the relative change in light intensity) obtained from three replicate experiments, reflecting the interactions between the anti-CA125 aptamer, the interfering proteins, and the CA125 antibody. The assay results revealed that the mean relative light intensity change of the sandwich complex formed by the interaction of the anti-CA125 aptamer, CA125, and CA125 antibody was 665.83 a.u. This signal intensity confirms that the anti-CA125 aptamer binds specifically to CA125 and forms a stable sandwich structure with the CA125 antibody, thereby amplifying the detection signal. In contrast, when interfering proteins such as hIgG, hIgE, AFP, and CA153 were substituted for CA125, the signal intensity was significantly lower than that of the CA125 experimental group, with their respective mean relative light intensity changes remaining below 35 a.u. It suggests that the interfering proteins could not form an effective sandwich structure with the anti-CA125 aptamer and CA125 antibody, thus failing to generate a comparable experimental signal. Therefore, the presence of these interfering proteins does not notably impact the experimental results, even in complex biological samples. In conclusion, the experimental findings demonstrate that the sandwich structure detection method, utilizing the anti-CA125 aptamer and CA125 antibody, exhibits high specificity for CA125. Through comparative experiments with interfering proteins, we have shown that this method can efficiently distinguish CA125 from other proteins, exhibiting strong anti-interference capability and a high detection signal. This approach is not only suitable for the detection of CA125 but also provides a robust technical framework for the detection of other similar biomolecules.

### 3.4. Gradient Detection of CA125 Using Sandwich Method

**Figure 4:**
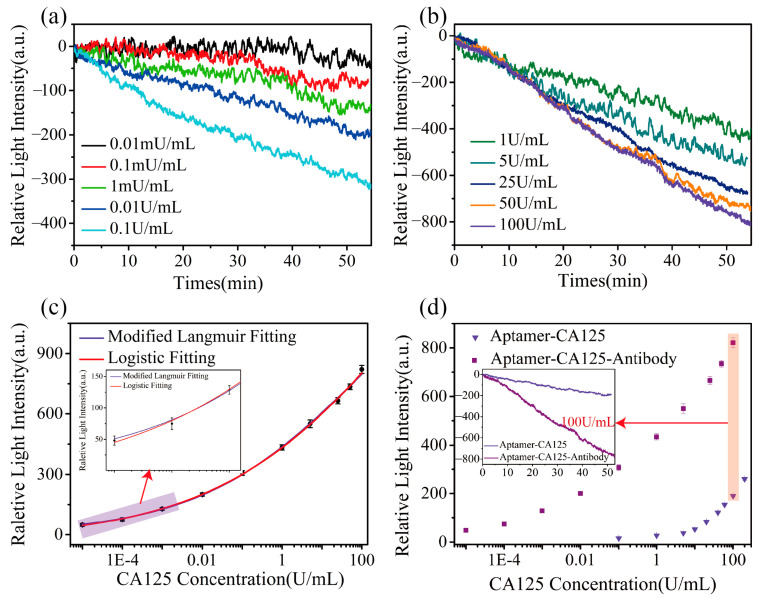
(**a**) Changes in relative light intensity for detecting different CA125 concentrations within the range of 0.01 mU/mL to 0.1 U/mL. (**b**) Detection results showing relative light intensity changes for CA125 concentrations ranging from 1 U/mL to 100 U/mL. (**c**) Fitting results using the modified Langmuir adsorption model (purple) and the logistic model (red), with the *x* axis (concentration) logarithmically transformed for clarity. (**d**) Comparative detection results of the sandwich method (aptamer–CA125–antibody) versus the direct method (aptamer–CA125). The inset illustrates the real-time detection curves for both methods at a CA125 concentration of 100 U/mL. The horizontal axis represents time (min), and the vertical axis represents the change in relative light intensity (a.u.).

In this study, we conducted a gradient detection of CA125 concentrations ranging from 0.01 mU/mL to 100 U/mL using a sandwich module with the anti-CA125 aptamer and CA125 antibody. PBS+ (10 mM PBS, containing 5 mM MgCl_2_) solution was used as the reference channel. One of the experimental results, presented in Figure 4, shows the corresponding average relative light intensity changes after the reaction with various concentrations of CA125, which were 48.09 a.u., 74.83 a.u., 128.46 a.u., 200.10 a.u., 307.07 a.u., 433.73 a.u., 550.08 a.u., 665.83 a.u., 735.11 a.u., and 821.22 a.u., respectively. Considering the noticeable differences in the results of the 10 experiments conducted in the manuscript, we grouped the data and created two separate plots to more clearly illustrate the experimental results, enabling a better understanding of the detection trends and the effectiveness of the method. As shown in Figure 4a,b, when CA125 is captured by the anti-CA125 aptamer and CA125 antibody, forming the sandwich complex, it induces a change in the refractive index of the chip surface. This change is ultimately reflected as a variation in light intensity, resulting in a shift in the curve. The curves represent the relative change in light intensity due to the binding reaction of CA125 at different concentrations. Specifically, at the lowest concentration (0.01 mU/mL), the experimental signal could be distinctly separated from the noise, and the intensity of the signal increased with higher concentrations. As the concentration of CA125 increased, the signal response, represented by the relative change in light intensity, demonstrated a clear concentration-dependent trend. The higher concentrations of CA125 resulted in a more considerable relative change in light intensity. Although the overall reaction did not reach saturation, repeated experiments showed that the relative light intensity change reached a stable value after approximately 50–60 min. Considering the potential influence of the method in practical applications, we chose to measure the relative light intensity change at 55 min into the reaction as experimental statistics. This value was then used to calculate the detection resolution. To ensure the method’s reliability, we verified the reproducibility of the experiments by repeating each concentration gradient experiment three times. All the results demonstrated the high precision and stability of the method.

In this study, different analytical approaches were employed to achieve specific objectives. For analyzing the kinetic parameters of intermolecular binding, the real-time kinetic experimental data were processed using a modified Langmuir fitting model to extract the kinetic parameters. Meanwhile, a four-parameter fitting model was utilized for quantitative analysis and to construct a standard curve to quantify unknown sample concentrations. The two distinct fitting results are illustrated in Figure 4c. The modified Langmuir fitting curve is described by the equation: $I=(1604.80*0.37*C^{0.21})/(1+0.37*C^{0.21})$, where I represents the response relative light intensity (a.u.), and *C* denotes the analyte CA125 concentration (U/mL). The R^2^ value of the modified Langmuir fitting curve is approximately 99.90%. The modified Langmuir model emphasizes the molecular binding affinity and the nonlinear concentration relationship, effectively describing the relationship between molecular recognition and sensor response.

The logistic fitting is a widely used nonlinear regression method that facilitates the generation of a standard curve correlating the concentration with response values. On the one hand, this approach evaluates the sensor’s sensitivity and response behavior across different concentrations, offering critical insights for subsequent experimental design and practical applications. On the other hand, the standard curve enables the accurate determination of the concentration of unknown samples. The logistic fitting equation for this experiment is $I=1604.89-(\frac{1604.89}{1+({\frac{C}{105.91})}^{0.21}})$, where I and *C* have the same meaning as above. The goodness of fitting (*R*^2^ = 99.90%) demonstrates a high level of consistency between the curve and the experimental data. Both fitting methods showed high data matching, proving the fitting methods’ feasibility.

The system exhibited a standard deviation (δ_T_) of 6.38 a.u. under stabilized conditions, which serves as a threshold to distinguish the changes in relative light intensity due to the CA125 concentration from system fluctuations. For accuracy, light intensity changes exceeding three times this standard deviation (19.14 a.u.) can be attributed to the specific interaction of CA125 with its aptamer and antibody. The sensor’s resolution for CA125 was determined as Cr = 3δ_T_/(ΔI/Δ*C*), where Cr represents the resolution, δT is the system’s standard deviation, and ΔI and Δ*C* are the changes in light intensity and concentration, respectively. With this formula, the resolution in PBS solution was calculated to be 3.98 μU/mL. Despite minor variations among experimental groups, the errors remained within a controllable range, demonstrating that the method reliably detects CA125 across low and high concentrations with minimal experimental error.

Additionally, we conducted direct detection experiments where anti-CA125 recognized CA125. The CA125 concentrations used were 0.1, 1, 5, 10, 20, 40, 60, 100, and 200 U/mL, with the corresponding relative light intensity changes measured as 14.86 a.u., 26.59 a.u., 37.24 a.u., 52.00 a.u., 85.90 a.u., 119.04 a.u., 151.22 a.u., 193.74 a.u., and 259.39 a.u., respectively. Specific comparisons between the two methods are illustrated in Figure 4d. To better observe the variations at low concentrations, the *x* axis was logarithmically scaled (log10) to display the relative light intensity changes induced by concentration differences. The experimental results demonstrate that the sandwich method significantly enhances signal detection and improves detection resolution. The inset shows the real-time detection curves of aptamer–CA125 complexes (direct method) and aptamer–CA125–antibody complexes (sandwich method) using the imaging weak measurement system at a CA125 concentration of 100 U/mL. The results clearly show that the relative light intensity changes obtained using the sandwich method are at least four times higher than those observed with the direct method, both at low concentrations and in high concentration ranges. These findings further highlight that the sandwich method offers a broader detection range and improved resolution, significantly enhancing the method’s specificity and sensitivity.

### 3.5. Detection Experiments of Different Sequence Aptamers

**Figure 5:**
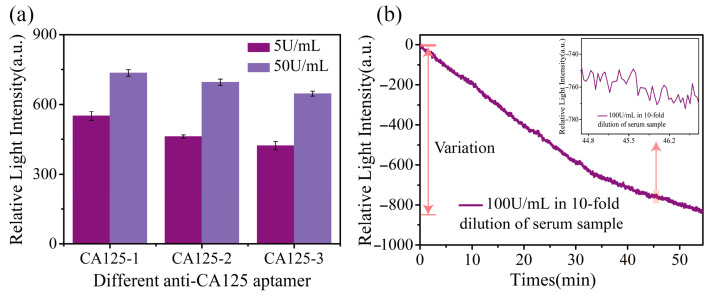
(**a**) Detection of CA125 using three different sequences of aptamers in sandwich assays, with fuchsia representing a CA125 concentration of 5 U/mL and purple representing a concentration of 50 U/mL. (**b**) Sandwich assay of CA125 in serum samples at 100 U/mL, with the inset showing fluctuations in the real-time monitoring curve.

In this study, three anti-CA125 aptamers with different sequences (CA125-1, CA125-2, and CA125-3) were designed, all at a concentration of 2 μM, to evaluate their detection ability for two different concentrations of CA125 (5 U/mL and 50 U/mL). The experimental results, shown in Figure 5a, demonstrated that, despite the differences in aptamer sequences, all three aptamers produced reliable detection signals for both CA125 concentrations. It suggests that the detection method is not sequence-dependent, highlighting its robustness and versatility across different aptamer sequences. In the high-concentration CA125 assay (50 U/mL), all three aptamers exhibited a notable light intensity variations, with the strongest signal observed for CA125-1 (735.11 a.u.). However, CA125-2 and CA125-3 also generated effective signals at 695.29 a.u. and 646.63 a.u., respectively. In contrast, the change in light intensity for the low-concentration CA125 assay (5 U/mL) was less than that produced by 50 U/mL but was still a very significant result. CA125-1 still showed the most pronounced response (550.08 a.u.), while CA125-3 gave the weakest response (422.52 a.u.). These results indicate that although the binding efficiency may vary between the different aptamers, all three provide effective signaling responses in CA125 samples at the corresponding concentrations. The results illustrate the versatility of the assay, which is not dependent on a specific aptamer sequence. Such flexibility makes the method adaptable to various aptamer sequence combinations, ensuring stable assay performance. Building on the specific experimental results presented in Section 3.3, we demonstrate that the anti-CA125 aptamer sensor chip developed in this study not only ensures the detection specificity but is also insensitive to changes in the aptamer sequence, showcasing its broad applicability and robustness in biosensing applications. Additionally, this characteristic could be beneficial for applications in aptamer screening.

### 3.6. Detection of CA125 in Complex Samples

Serum, as a complex biological sample, contains many components, including proteins, lipids, and immune factors. In this experiment, we spiked a serum sample with CA125 protein at a concentration of 100 U/mL to generate a complex mixture containing both CA125 and the typical serum constituents. We then employed the method proposed in this study to detect CA125 in this adulterated sample. Figure 5b presents the results of this assay. We incorporated two reference channels to detect complex samples: one for serum and one for PBS+ solution. The experimental flow channel introduced the serum sample spiked with CA125. The change in the response value of the PBS part of the experimental and reference channels is used as the raw data for calibration, and the differential processing through the experimental and reference flow channels utilizes the feature that PBS does not cause changes in light intensity to ensure the accuracy of the results. By sequentially subtracting the human serum and PBS+ reference from the experimental result (CA125 + human serum), the real-time binding curve specific to 100 U/mL of CA125 in a complex sample assay is obtained, as shown in Figure 5b. Adding 100 U/mL of CA125 to human serum induced a relative light intensity change of 829.1 a.u. These results demonstrate that the proposed method effectively recognizes and quantitatively detects CA125, producing a signal significantly above the background noise, thus validating the method’s effectiveness and feasibility for detecting CA125 in complex biological samples.

As illustrated in Table 1, a comparative analysis of the detection accuracy of different biosensors for CA125 was conducted. Notably, the method employed in this study exhibited a distinct advantage in detection accuracy compared to other methods reported in the literature.

## 4. Conclusions

In this study, we present a novel aptamer–antibody sandwich assay integrated with an imaging weak measurement system to detect biomolecules. This method leverages the synergistic recognition capabilities of aptamers and antibodies, enabling the efficient capture and sensitive detection of CA125. The experimental results demonstrated that the proposed method exhibited exceptional sensitivity and high resolution across a concentration range of 0.01 mU/mL to 100 U/mL, coupled with robust anti-interference capabilities. Notably, the method’s performance remained unaffected by variations in aptamer sequences. Furthermore, detecting CA125 in serum samples confirmed the method’s applicability to complex biological matrices. In comparative analyses, the relative light intensity changes obtained using the sandwich assay were at least four times greater than those observed with the direct method (anti-CA125 aptamer recognition of CA125), highlighting the enhanced signal amplification inherent in the sandwich approach. The detection resolution of the proposed method in PBS solution was determined to be 3.98 μU/mL, which is significantly lower than that achieved by the direct method. Compared with existing detection techniques, the proposed method offers significant advantages, including high sensitivity, specificity, label-free, and real-time monitoring capabilities, providing a powerful platform for molecular detection with great potential in different applications, especially in clinical diagnostics, environmental monitoring, and biological research.

## Figures and Tables

**Table 1:** The resolution of CA125 detection by different methods, previously reported in the literature.

Detection Method	Receptor	Measurement Range	Resolution	Ref.
Surface plasmon resonance	Aptamer	10–100 U/mL	0.01 U/mL	[17]
Electrochemical	Aptamer	0.0001–500 U/mL	0.1 mU/mL	[43]
Surface plasmon Resonance imaging	Antibody	2.2–150 U/mL	0.66 U/mL	[44]
Fluorescent and magnetic MIP	Antibody	0.0005–40 U/mL	50 μU/mL	[45]
Electrochemical affinity sensor	Aptamer, antibody	2–100 U/mL	0.08 U/mL	[46]
Fluorescence resonance Energy transfer	Aptamer, antibody	1.0 μU/mL–1.0 U/mL	0.5 μU/mL	[47]
3D carbon nanotube network biochip	Aptamer, antibody	10 mU/mL–1000 U/mL	10 mU/mL	[48]
WVA-based sandwich method	Aptamer, antibody	0.01 mU/mL–100 U/mL	3.98 μU/mL	This work

## Data Availability

Data will be made available upon request.

## Supplementary Materials

The following supporting information can be downloaded at https://www.mdpi.com/article/10.3390/bios15050268/s1, Figure S1: (a) 10-flow channel chip obtained by 3D printing; (b) Physical view of the detection chip used in the experiment (front view); (c) Physical view of the detection chip used in the experiment (top view). Figure S2: Use the system to monitor real-time response curves for different concentrations of NaCl solutions (0, 5, 10, 15, 20, and 25 g/mL). Figure S3: A display of an image captured in one of the experiments. References [49,50,51,52,53,54] are cited in the Supplementary Materials.

## Abbreviations

The following abbreviations are used in this manuscript:

hIgG	Human IgG
hIgE	Human IgE
AFP	Alpha-fetoprotein
PBS	Phosphate-buffered solution
ELISA	Enzyme-linked immunosorbent assays
