# Supplementary material for: Synergistic Signal Amplification via Weak Value Amplification Effect and Sandwich Structure for Highly Sensitive and Specific Real-Time Detection of CA125
Source: Biosensors (Basel). 2025 Apr 23;15(5):268. doi: 10.3390/bios15050268 (PMC12109719; doi:10.3390/bios15050268)
Supplement: Supplementary file 1 [file biosensors-15-00268-s001.zip › biosensors-3537883-supplementary.pdf]

# Synergistic Signal Amplification via Weak Value Amplification Effect and Sandwich Structure for Highly Sensitive and Specific Real-Time Detection of CA125

**Bei Wang [1,2], Yang Xu [3], Han Li [1,2], Zishuo Song [1,2], Tian Guan [1,2,*] and Yonghong He [1,2,*]**

[1] Shenzhen Key Laboratory for Minimal Invasive Medical Technologies, Institute of Optical Imaging and Sensing, Tsinghua Shenzhen International Graduate School, Tsinghua University, Shenzhen 518055, China
[2] Institute of Biopharmaceutical and Health Engineering, Tsinghua Shenzhen International Graduate School, Tsinghua University, Shenzhen 518055, China
[3] Department of Laboratory Medicine, Shenzhen Children's Hospital, Shenzhen 518038, China
* Correspondence: guantian@sz.tsinghua.edu.cn (T.G.); heyh@sz.tsinghua.edu.cn (Y.H.)

## S1.1 Theory

Weak measurement was first proposed by Aharonov, Albert, and Vaidman in 1988 within the framework of quantum mechanics, representing a significant extension of traditional quantum measurement theory. Unlike strong measurement, which induces wavefunction collapse and results in irreversible changes to the system's state, weak measurement employs a weakly coupled measuring device, minimizing perturbation to the system. The approach mitigates the disruptive effects of traditional measurement while enabling the exploration of quantum interference effects during the measurement process.

The primary distinction between weak and strong measurements lies in the strength of the coupling between the measuring device and the system under observation. In strong measurement, the results follow a classical probability distribution, with measured values strictly confined to the eigenvalue range, and the system collapsing into the corresponding eigenstate. In contrast, weak measurement incorporates pre- and post-selection, meaning that measurements are conducted only when specific pre- and post-selection conditions are met. The approach allows for the observation of weak values that extend beyond the conventional eigenvalue range. Such weak values do not directly correspond to the eigenvalues of physically observable quantities but instead emerge from the interplay between the system's initial state, the measurement operator, and the post-selected state.

In the realm of weak measurement technology, a key concept is the disturbance

relationship that exists between the physical parameters being measured and the measurement system itself. Specifically, weak measurement systems operate under weak coupling between the system and the pointer states. This weak interaction results in a subtle separation of eigenstates within the system, which is typically too small to detect by conventional measurement methods. However, a weak measurement system is designed to detect this minute separation by employing a highly sensitive technique. By carefully adjusting the pre- and post-selection polarization states, it is possible to amplify this separation, magnifying the observed signals by several orders of magnitude relative to the original eigenvalues. This amplification, referred to as the "weak value amplification effect". The weak value amplification effect is a significant application of weak measurement. By appropriately selecting the post-selected state, it enables the amplification of subtle signal variations to a measurable range, thereby enhancing the system's sensitivity to weak signals. This effect has been widely applied in high-precision measurement fields, including optical interferometry, nanoscale displacement detection, and biosensing technologies. Although the signal from a single measurement remains weak in weak value amplification, statistical processing of a large number of measurements allows for the effective extraction of key information, ultimately improving detection capabilities. In biomolecular detection, this principle is leveraged to achieve ultra-high sensitivity by exploiting the weak coupling and indirect measurement approach. It makes weak measurement technology a powerful tool for the detection of biomolecules, significantly enhancing the sensitivity and accuracy of measurements that would otherwise be undetectable using traditional methods.

This study uses a Gaussian representation for the light sources to maintain generality and simplicity. The frequency-domain wave function of the red LED light source is modeled as a Gaussian function, which can be expressed as follows:

$$(\omega|\xi) = f(\omega) = (\pi\Delta^2)^{-1/4} e^{-(w-w_0)^2/2\triangle^2} \qquad \text{(S1)}$$

The polarization direction of the front polarizer makes an angle of $\pi/4$ with the vertical direction, and the system state is denoted as $|\psi>=\frac{\sqrt{2}}{2}(|H\rangle+|V\rangle)$. Note that $|H\rangle$ and $|V\rangle$denote the horizontal and vertical linear polarization states, respectively.

The beam emitted by the light source is incident on the inner surface of the prism at an angle of incidence θ slightly larger than the critical angle. This process induces a phase difference between p- and s-light in the reflected light. According to Fresnel's theore

$$\varphi=2\,tan^{-1}\frac{\sqrt{n^2\,sin^2\,\theta-1}}{n\,sin\,\theta\,tan\,\theta} \tag{S2}$$

Here, n=n1/n2, where n1 is the refractive index of the prism (n1=1.73) and n2 is the equivalent refractive index of the object to be measured. Therefore, the state of the reflection system of the reflected light can be expressed as:

$$\frac{\sqrt{2}}{2}\left(e^{i\frac{\varphi}{2}}|H\rangle+e^{-i\frac{\varphi}{2}}|V\rangle\right) \tag{S3}$$

Subsequently, the beam passes through the achromatic quarter waveplate, the angle between the quarter waveplate axis and the vertical direction is π/4, at this time the beam and the waveplate incense interaction is expressed as $e^{i\frac{\pi}{4}(|H\rangle\langle V|-|V\rangle\langle H|)}$ . Therefore, the coupling state of the beam after preselection is summarized as follows:

$$\begin{aligned}\langle\omega|\psi_i\rangle|\xi\rangle &= (\pi\Delta^2)^{-\frac{1}{4}}e^{-\frac{(\omega-\omega_0)^2}{2\Delta^2}}e^{i\frac{\pi}{4}(|H\rangle\langle V|-|V\rangle\langle H|)}\frac{\sqrt{2}}{2}\left(e^{i\frac{\varphi}{2}}|H\rangle+e^{-i\frac{\varphi}{2}}|V\rangle\right)\\ &= \left(cos(\frac{\pi}{4}+\frac{\varphi}{2})|H\rangle+sin(\frac{\pi}{4}+\frac{\varphi}{2})|V\rangle\right)(\pi\Delta^2)^{-1/4}e^{-\frac{(\omega-\omega_0)^2}{2\Delta^2}}\end{aligned} \tag{S4}$$

In this paper, the weak interaction is realized by means of a quartz rotor. The coupling between the polarization state and the frequency domain can be expressed using the operator $U=e^{-i\tau A\omega}$, where τ is the coupling strength, which is related to the thickness of the rotator d. We use t when the thickness d=1 mm, $\omega$ is the optical frequency, and A is the polarization operator $A=-i|H\rangle\langle V|+i|V\rangle\langle H|$ . The state of the system after weak coupling can then be expressed as:

$$\begin{aligned}\langle\omega|\xi\rangle|\psi_i\rangle &= \left(sin(\frac{\pi}{4}+\frac{\varphi}{2})e^{\tau\omega}|H\rangle+cos(\frac{\pi}{4}\right.\\ &\left.+\frac{\varphi}{2})e^{-\tau\omega}|V\rangle\right)(\pi\Delta^2)^{-1/4}e^{-\frac{(\omega-\omega_0)^2}{2\Delta^2}}\end{aligned} \tag{S5}$$

In previous work, we have implemented high-precision measurements of other physical quantities measuring the offset of the center wavelength. Here, the post-selection process is realized by another polarizer, with the state expressed as $|\psi_f\rangle=sin(\frac{3\pi}{4}+\varepsilon)|H\rangle+cos(\frac{3\pi}{4}+\varepsilon)|V\rangle$ , where $\varepsilon\ll 1$, the front and back polarization

states are nearly orthogonal. According to the weak measurement theory, it is known that the amplification coefficient of the displacement of the center wavelength is related to the weak value.

The weak value expression is:

$$A_{\omega} = \frac{\langle\psi_f|A|\psi_i\rangle}{\langle\psi_f|\psi_i\rangle} = i\frac{1}{\varepsilon - \frac{\varphi}{2}} \tag{S6}$$

When $\varepsilon, \varphi \ll 1$, the post-selection process amplifies the center wavelength shift. The inverse weak value mechanism is usually used as the working mechanism due to its excellent amplification performance. However, WVA can also be realized in the $\tau\omega_0 < \left|\varepsilon - \frac{\varphi}{2}\right| < 1$ interval is realized. Therefore, using the working state, the relationship between the relative light intensity and the phase difference φ can be expressed as

$$\begin{aligned} I = \langle\omega|\xi\rangle\langle\psi_f|\psi_i\rangle = \int d\omega\, sin(\tau\omega + \varepsilon - \frac{\varphi}{2})(\pi\Delta^2)^{-\frac{1}{4}} e^{-\frac{(\omega-\omega_0)^2}{2\Delta^2}} \\ =1\text{-}e^{-\Delta^2\tau^2} cos\, 2\left(\tau\omega_0 + \varepsilon - \frac{\varphi}{2}\right) \\ \approx 2\sin^2(\tau\omega_0 + \varepsilon - \frac{\varphi}{2}) \ll 1 \end{aligned} \tag{S7}$$

The relationship between the light intensity and the phase φ of the post-selected state can be obtained according to the above equation, and an approximately linear relationship can be obtained when measured in a smaller range. In our experiments, we use the above range as our detection interval.

## S1.2 Experimental Preparation and Pre-testing

Before starting the experiment, the ZF6 prism and the 3D-printed 10-fluid channel chip were bonded using AB glue and left at room temperature for 24 hours to ensure chip stability. The dimensions of the prism were 27 mm × 27 mm × 20 mm, while the 3D-printed channel measured 27 mm × 20 mm × 5 mm, with each channel approximately 10 mm × 1.5 mm × 0.8 mm (schematically shown in Figure S1). According to the experimental design, at least two adjacent flow channels were pre-selected: one served as the experimental channel and the other as the reference channel. The data processing in this study employs a self-reference pixel-averaging method. Specifically, the signal from the experimental channel on the same chip is subtracted

from the signal of the reference channel. This approach effectively corrects system errors, eliminates background noise, and monitors experimental stability. Moreover, it isolates the target signal, thereby enhancing the detection's sensitivity, specificity, and reliability.

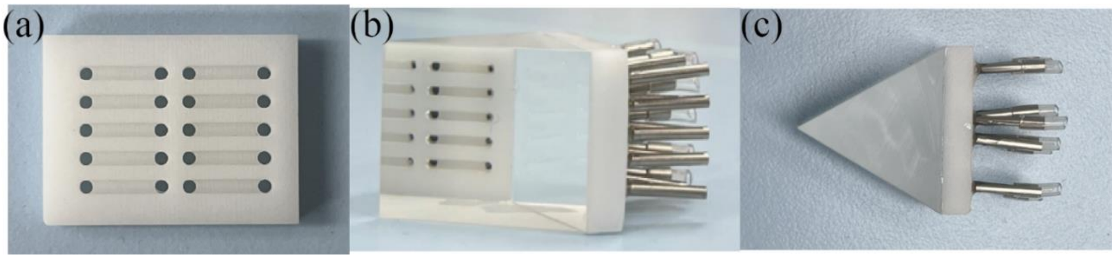


**Figure S1**. (a) 10-flow channel chip obtained by 3D printing; (b) Physical view of the detection chip used in the experiment (front view); (c) Physical view of the detection chip used in the experiment (top view).

Depending on the experimental requirements, a standard solution (e.g., deionized water, phosphate buffer, or other buffers) was sequentially passed into both channels. Experimental images were then captured to calculate the light intensity changes in the two channels, enabling correction of the experimental data. Differential processing of image data from the experimental and reference channels was performed to eliminate the effects of temperature fluctuations, non-specific signal interference, and other artifacts, ensuring the accuracy of the results.

Captured images were 16-bit with a maximum light intensity of 65536 a.u. During system calibration, the light intensity of the phosphate buffer solution (PBS) was maintained at approximately 33,000 a.u., a level optimized during system debugging prior to the main experiment. To validate the system’s stability, NaCl solutions of varying concentrations (0, 5, 10, 15, 20, and 25 g/mL) were tested, confirming that the detectable light intensity changes remained within a relatively stable range, as Fig. S2 shows.

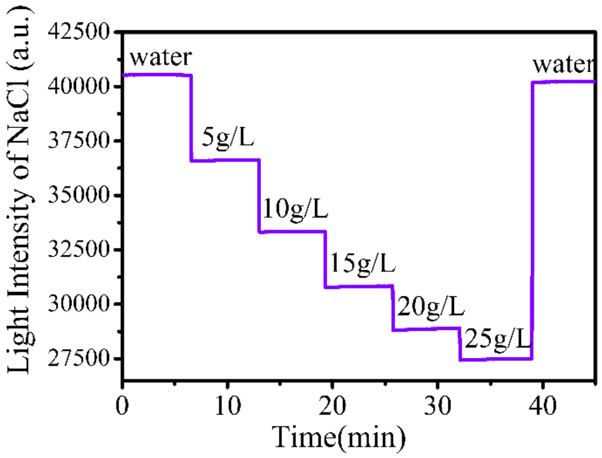


**Figure S2**. Use the system to monitor real-time response curves for different concentrations of NaCl solutions (0, 5, 10, 15, 20, and 25 g/mL).

## S2.1 Experimental materials

The reagents and consumables used in this experimental species are shown below. Alpha-fetoprotein (AFP), glycoprotein CA153, glycoprotein CA125 and CA125 antibody were purchased from Beijing Zhipeng Co., Ltd. (Beijing, China). Human immunoglobulin E (IgE), human immunoglobulin G (IgG) and bovine serum albumin (BSA) were purchased from Beijing Bioss Technology Co., Ltd. (Beijing, China). Sodium chloride (NaCl), Magnesium chloride (MgCl2), dopamine hydrochloride and tris(hydroxymethyl)aminomethane (Tris) were purchased from Aladdin Co., Ltd. (Shanghai, China). Phosphate buffer solution (PBS) powder and human serum were purchased from Beijing Solarbio Technology Co., Ltd (Beijing, China). Blocking solution (2%) and Tris buffer (low EDTA) were purchased from Shanghai Sangon Biotech Co., Ltd. (Shanghai, China). ZF6 prisms were brought from Fuzhou Alpha Optical Co., Ltd. (Fuzhou, Fujian, China).

The anti-CA125 aptamer sequence was synthesized by Sangon Biotech, Shanghai, China, and the specific sequence includes:

| Name | Sequence |
|---|---|
| CA125-1 | 5'-NH2-CTCACTACTATAGGGATAGGGAGACAAGAATAAACG CTCAA-3' |
| CA125-2 | 5'-NH2-C6H12-TTATCGTACGACAGTCATCCTACAC-3' |
| CA125-3 | 5'-NH2-C6H12-TATCAATTACTTACCCTAGTGGTGTGATGTCG TATGGATG-3' |

The specificity experiments, gradient experiments, concentration optimization experiments for aptamer use, and detection experiments in serum samples in this paper are all based on CA125-1.

Based on literature research and the experience of previous researchers in our group, this thesis does not include detailed studies on the optimization of certain fundamental experimental conditions, such as the adjustment of dopamine concentration [1-3], the immobilization of the anti-CA125 aptamer and its interaction with the CA125 protein [4,5], and the specific binding of the CA125 protein to its antibody [6,7]. Instead, the primary focus of this work was the optimization of aptamer concentration and aptamer sequence.

### S2.2 Optimization of anti-CA125 aptamers on the chip surface

The optimization of anti-CA125 aptamer concentration was initially carried out following the procedure outlined below. First, the flow channel was cleaned using 0.01 M PBS solution to ensure the channel was free of contaminants and to remove any dust particles. Next, a 0.01 M Tris buffer was prepared, and a 1 g/L dopamine-Tris solution (pH 8.5) was prepared using this buffer. The dopamine-Tris solution was introduced into the flow channel and allowed to react for 20 minutes. In the alkaline environment, dopamine undergoes self-polymerization to form polydopamine, which then self-assembles onto the prism surface, creating a reactive layer with functional groups such as catechols and amino groups.

After 20 minutes, the channel was flushed with 0.01 M PBS solution to remove residual dopamine-Tris solution. Subsequently, the PBS-cleaned flow channel was sequentially injected with solutions of aminocarbamate-anti-CA125 aptamer at concentrations of 0.25, 0.5, 1, 2, and 5 μM at a flow rate of 38 μL/min. The reaction proceeded for 60 minutes, while the reference channel was continuously supplied with PBS solution. Following this, the flow channel was washed with PBS and then treated with a blocking solution for 60 minutes to close unoccupied sites. After an additional 40-minute wash with PBS, anti-CA125 aptamer microarrays were formed at different concentrations.

For the detection phase, CA125 (prepared in PBS+ solution) at a concentration of 100 U/mL was introduced into both the experimental and reference channels, where the reaction continued for 60 minutes at a flow rate of 38 μL/min. The channels were then

washed with PBS to remove any unbound CA125. Subsequently, the CA125 antibody was injected into the flow channels at a concentration of 5 μg/mL (concentration determined by theoretical calculations and experimental experience). After a final wash with PBS to remove unbound antibodies, the system was stabilized with PBS for 30 minutes. The experimental data were processed and analyzed using MATLAB.

### S2.3 Specificity experiments with different concentrations of CA125

The experiments were conducted according to the procedure outlined in Fig. 1. The concentration of the anti-CA125 aptamer was determined based on the experimental results in section 2.2. The basic experimental steps closely followed the previously described protocol. Initially, the chip modified with anti-CA125 aptamers was prepared. PBS buffer was sequentially injected into the flow channels, and the reaction was carried out for 40 minutes. During this period, each channel's average change in light intensity response was recorded, serving as the background noise signal for the system. Once the system reached a stable state, the CA125 solution was introduced into the experimental flow channel, while the reference flow channel was supplied with PBS+ (0.1 M PBS solution containing 5 mM $MgCl_2$) buffer. The reaction continued for 60 minutes, after which PBS solution was introduced for 60 minutes. This step served both to remove any unreacted CA125 and to test the stability of the system. Subsequently, CA125 antibody solution at a 5 μg/mL concentration was introduced into the flow channels. After 60 minutes of reaction, PBS solution was passed through the channels to remove any unbound CA125 antibody. The experiment concluded after a final stabilization period of 60 minutes with PBS solution.

The weak measurement system continuously recorded the changes in light intensity for the experimental and reference flow channels in real-time. The CA125 solution was prepared by diluting it with PBS+ buffer to achieve concentrations of 0.01 mU/mL, 0.1 mU/mL, 1 mU/mL, 0.01 U/mL, 0.1 U/mL, 1 U/mL, 5 U/mL, 25 U/mL, 50 U/mL, and 100 U/mL. Upon completion of the experiments, the collected data were processed using MATLAB.

Four interfering proteins—human IgG, IgE, AFP, and CA153—were used for the specificity validation experiments. The human IgG, IgE, and AFP concentrations were set at 25 ng/mL, while the concentration of CA153 was 25 U/mL. All experimental conditions, except for the reaction proteins, were maintained consistent with those used

in the gradient experiments. The data collection and processing procedures were also identical.

### S2.4 Anti-CA125 aptamer detection experiments with different sequences

To investigate whether the proposed method is influenced by variations in aptamer sequences, three distinct anti-CA125 aptamers were designed. These sequences were designated as CA125-1, CA125-2, and CA125-3. The concentration of each aptamer was set at 2 μM, and CA125 concentrations of 5 U/mL and 50 U/mL were tested. All experimental procedures and conditions were consistent across the tests, and the data collection and processing methods were also standardized.

### S2.5 Detection of CA125 in complex samples

For the human serum sample experiments, three flow channels were utilized: one experimental channel and two reference channels. The reference channels were filled with PBS+ solution and human serum solution, respectively, to account for potential interference and ensure the accuracy of the experimental results. Commercially purchased human serum was diluted 10-fold and spiked with 100 U/mL of CA125, creating adulterated samples for testing. All other experimental conditions were consistent with those used in the PBS+ solution experiments. During the sample detection step, the prepared spiked serum samples were introduced into the experimental channel at a constant flow rate, allowing the anti-CA125 aptamer to bind specifically to the CA125 in the samples. The aptamer then formed a complex with the CA125 antibody, creating a sandwich structure for detection. Data analysis was conducted using Matlab.

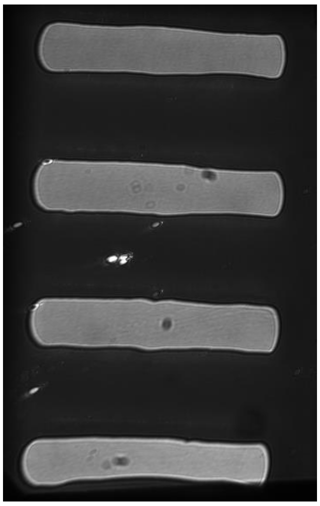

**Figure S3**. A display of an image captured in one of the experiments.

## References


1. Lee, H.; Dellatore, S.M.; Miller, W.M.; Messersmith, P.B. Mussel-Inspired Surface Chemistry for Multifunctional Coatings. Science 2007, 318, 426-430, doi:10.1126/science.1147241.
2. Liu, Y.; Ai, K.; Lu, L. Polydopamine and Its Derivative Materials: Synthesis and Promising Applications in Energy, Environmental, and Biomedical Fields. Chemical Reviews 2014, 114, 5057-5115, doi:10.1021/cr400407a.
3. Bernsmann, F.; Ponche, A.; Ringwald, C.; Hemmerlé, J.; Raya, J.; Bechinger, B.; Voegel, J.-C.; Schaaf, P.; Ball, V. Characterization of Dopamine−Melanin Growth on Silicon Oxide. The Journal of Physical Chemistry C 2009, 113, 8234-8242, doi:10.1021/jp901188h.
4. Gedi, V.; Song, C.K.; Kim, G.B.; Lee, J.O.; Oh, E.; Shin, B.S.; Jung, M.; Shim, J.; Lee, H.; Kim, Y.-P. Sensitive on-chip detection of cancer antigen 125 using a DNA aptamer/carbon nanotube network platform. Sensors and Actuators B: Chemical 2018, 256, 89-97, doi:https://doi.org/10.1016/j.snb.2017.10.049.
5. Tripathi, P.; Kumar, A.; Sachan, M.; Gupta, S.; Nara, S. Aptamer-gold nanozyme based competitive lateral flow assay for rapid detection of CA125 in human serum. Biosensors and Bioelectronics 2020, 165, 112368, doi:https://doi.org/10.1016/j.bios.2020.112368.
6. Mandal, D.; Nunna, B.B.; Zhuang, S.; Rakshit, S.; Lee, E.S. Carbon nanotubes based biosensor for detection of cancer antigens (CA-125) under shear flow condition. Nano-Structures & Nano-Objects 2018, 15, 180-185, doi:https://doi.org/10.1016/j.nanoso.2017.09.013.
7. Baradoke, A.; Jose, B.; Pauliukaite, R.; Forster, R.J. Properties of Anti-CA125 antibody layers on screen-printed carbon electrodes modified by gold and platinum nanostructures. Electrochimica Acta 2019, 306, 299-306, doi:https://doi.org/10.1016/j.electacta.2019.03.081.
